# Enzymatic, Antioxidant, and Antimicrobial Activities of Bioactive Compounds from Avocado (*Persea americana* L.) Seeds

**DOI:** 10.3390/plants12051201

**Published:** 2023-03-06

**Authors:** Kaja Kupnik, Mateja Primožič, Vanja Kokol, Željko Knez, Maja Leitgeb

**Affiliations:** 1 Faculty of Chemistry and Chemical Engineering, University of Maribor, Smetanova Ulica 17, 2000 Maribor, Slovenia; 2 Faculty of Mechanical Engineering, University of Maribor, Smetanova Ulica 17, 2000 Maribor, Slovenia; 3 Faculty of Medicine, University of Maribor, Taborska Ulica 8, 2000 Maribor, Slovenia

**Keywords:** avocado seed, *Persea americana*, HPLC, phenolic compounds, enzymatic activity, phytochemistry, antioxidant, antimicrobial activity

## Abstract

The aim of this research was to identify and quantify biologically active compounds from avocado (*Persea americana* L.) seeds (AS) utilizing different techniques with the use of ultrasound (US), ethanol (EtOH), and supercritical carbon dioxide (scCO_2_) for possible applications in (bio)medicine, pharmaceutical, cosmetic, or other relevant industries. Initially, a study of the process efficiency (*η*) was carried out, which revealed yields in the range of 2.96–12.11 wt%. The sample obtained using scCO_2_ was found to be the richest in total phenols (TPC) and total proteins (PC), while the sample obtained with the use of EtOH resulted in the highest content of proanthocyanidins (PAC). Phytochemical screening of AS samples, quantified by the HPLC method, indicated the presence of 14 specific phenolic compounds. In addition, the activity of the selected enzymes (cellulase, lipase, peroxidase, polyphenol oxidase, protease, transglutaminase, and superoxide dismutase) was quantified for the first time in the samples from AS. Using DPPH radical scavenging activity, the highest antioxidant potential (67.49%) was detected in the sample obtained with EtOH. The antimicrobial activity was studied using disc diffusion method against 15 microorganisms. Additionally, for the first time, the antimicrobial effectiveness of AS extract was quantified by determination of microbial growth-inhibition rates (MGIRs) at different concentrations of AS extract against three strains of Gram-negative (*Escherichia coli*, *Pseudomonas aeruginosa*, and *Pseudomonas fluorescens*) bacteria, three strains of Gram-positive (*Bacillus cereus*, *Staphylococcus aureus*, and *Streptococcus pyogenes*) bacteria, and fungi (*Candida albicans*). MGIRs and minimal inhibitory concentration (MIC_90_) values were determined after 8 and 24 h of incubation, thus enabling the screening of antimicrobial efficacy for possible further applications of AS extracts as antimicrobial agents in (bio)medicine, pharmaceutical, cosmetic, or other industries. For example, the lowest MIC_90_ value was determined for *B. cereus* after 8 h of incubation in the case of UE and SFE extracts (70 μg/mL), indicating an outstanding result and the potential of AS extracts, as the MIC values for *B. cereus* have not been investigated so far.

## 1. Introduction

Avocado (*Persea americana* L.) is a nutritious tropical fruit belonging to the *Lauraceae* family and is known also as alligator pear. Due to its taste and texture and medicinal and nutritional properties, avocado has become indispensable on menus around the world, which has increased its production over the last decade. Its higher direct consumption and industrial processing is reflected in its fastest and highest production growth in 2020 and in previous years among tropical fruits, especially for the most popular Hass variety [1]. Accordingly, avocado seeds (AS), which are underutilized as inedible part and therefore discarded, can account for up to 26% of the total fruit weight [2] and represent large amounts of waste biomass but are, on the other hand, an inexpensive alternative source of potential bioactive compounds that remain unrecovered. 

In addition to the exceptional nutritional quality of avocado fruits, the chemical profile of various AS extracts comprises a high content of phytochemicals exhibiting a variety of biological activities. These include polyphenols, triterpenoids, acetogenins, fatty acids, and other compounds that have resulted in various properties and are beneficial to health because of their antihypertensive, antimicrobial, antioxidant, larvicidal, and hypolipidemic activity [3,4,5]. For example, aqueous AS extract showed a reduction in low-density lipoprotein cholesterol, total cholesterol, and triglycerides levels in hypertensive rats and also reduced their blood pressure [6], while aqueous AS extract reduced hyperglycemia in adult male rabbits [7]. AS extracts are also promising in the management of diabetes mellitus, as blood glucose levels in rats were significantly decreased because of antihyperglycemic and hypoglycemic activities [8], and inhibitory activities on some type 2 diabetes enzymes (α-glucosidase and α-amylase) have been also demonstrated [9]. Additionally, avocado leaves and seeds are also used in traditional medicine for the management of Alzheimer’s disease, which was also supported by an in vitro study that showed antioxidant and anti-cholinesterase activities [10]. The in vitro cytotoxic properties of AS against divergent types of cancer cell lines have also been reported in the literature [11,12,13] and have been examined in preclinical animal models [14]. 

For retrieval of the mentioned phytochemicals and biologically active substances from AS, mainly traditional and conventional methods have been used, including maceration, Soxhlet extraction (SE), and steam distillation, as these methods are easy to use and are well established. Nevertheless, these methods are time-consuming, require large amounts of organic solvents, and operate at high temperatures. Consequently, this leads to higher emissions of volatile organic solvents, can result in degradation of thermo-labile compounds, and significantly reduce the biological activity of the obtained active ingredients [15]. These shortcomings have recently prompted studies of more efficient, advanced, and green extraction techniques including ultrasonic (UE) [16], enzyme-assisted [17], cold-pressed [18], microwave [19], and supercritical fluid (SFE) extraction [20,21].

Among the above-mentioned extraction techniques, UE is one of the emerging methods, as it often provides a high extraction yield, uses mild temperatures, and is simple and inexpensive. Due to the frequency of ultrasonic irradiation, vibration occurs between matrix molecules, which leads to cavitation and, consequently, by creating macroturbulences and high-velocity inner-particle collisions, to easier leaching of bioactive compounds from the plant matrix into the solvent [22]. On the other hand, SFE with carbon dioxide (scCO_2_) as a solvent provides separation at near-ambient temperatures, therefore minimizing the degradation of thermo-labile compounds. Moreover, due to its green process (e.g., solvent-free products, short extraction time, low operating temperature, high extract quality, environmental friendliness, simplicity, etc.) and the great characteristics of scCO_2_ (e.g., innocuous to environment and human health, non-toxicity, mild critical temperature, low critical pressure, non-flammability, odorless nature, prevent extract oxidation, easily volatilized, etc.), it is one of the most promising techniques in the recovery of pure and clean extracts with bioactive compounds that can be used in biomedicine and for cosmetics, pharmaceutical products, nutraceuticals, and functional food [22,23]. It should be noted that a very limited number of studies using SFE for extraction of biologically active compounds from AS has been found in the literature. Hence, this investigation focuses on phytochemicals and bioactive compounds derived from AS and compares well-established UE and SE to the sustainable extraction method of SFE, which has never been done before. The findings of this study present useful information and tools, as avocado by-products from industrial processing could be used to generate a functional, high-value-added product with biological activity through efficient, feasible, and sustainable extraction processes.

The content of bioactive compounds and overall yield are vigorously influenced by the method of extraction and by the choice of solvent. Therefore, the presented research emphasizes the comparison of different extraction techniques (UE, SE, and SFE), process conditions (temperature and time), and solvents (H_2_O, EtOH, and CO_2_ with EtOH as co-solvent) used on the production yield of phytochemicals and high-value-added bioactive compounds such as phenols (TPC), proanthocyanidins (PAC), proteins (PC), and specific polyphenols (i.e., flavonoids and phenolic acids) in the AS extracts. A major contribution and the importance of our research is in the comparison of SFE results with the results obtained from the remaining, more widely used extraction methods, namely UE and SE. To the best of our knowledge, and after reviewing the literature, the content of certain polyphenols in SFE AS extracts has not yet been reported. This is also the first study in which various enzymatic activities (α-amylase, cellulase, lipase, peroxidase, protease and transglutaminase, polyphenol oxidase, and superoxide dismutase) have been determined in UE, SE, and SFE AS extracts and compared. In addition to the various enzymatic activities, the antioxidant and antimicrobial activity of UE, SE, and SFE extracts were also evaluated and compared. Antimicrobial activity was determined against 15 microorganisms including Gram-negative, Gram-positive bacteria, and fungi. Additionally, the antimicrobial activity of the extracts was quantified after 8 and 24 h of incubation by determination of microbial growth-inhibition rates (MGIRs) at five different concentrations and by determination of MIC_90_ value, which, to the best of our knowledge, has not yet been so thoroughly studied. Our study provides a comprehensive insight and new information on the recovery of bioactive compounds from AS, which could be potentially used in cosmetics, functional food, nutraceuticals, pharmaceutical products, and (bio)medicine. 

## 2. Results and Discussion

### 2.1. Effect of Changing Solvent and Techniques on Process Efficiency

**Figure 1:**
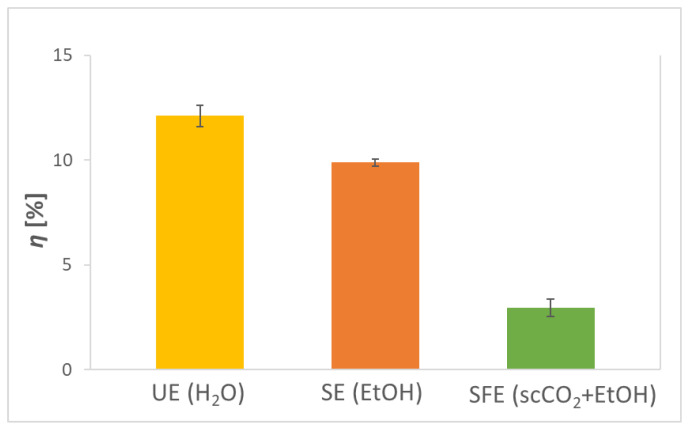
Extraction yields (*η*) obtained by ultrasonic extraction (UE; solvent H_2_O), Soxhlet extraction (SE; solvent EtOH), and supercritical fluid extraction (SFE; solvent scCO_2_, co-solvent EtOH) of avocado seed (AS).

The results in Figure 1 represent the extraction yield and thus indicate the efficiency of the performed AS extractions using three different methods (UE, SE, and SFE). UE provided the highest yield with 12.11%, followed by SE (9.88%) and SFE (2.96%). There was also a statistically significant difference between extraction yields as determined by one-way ANOVA. A Tukey’s post hoc test revealed that the extraction yield was statistically significantly lower using SFE (*p* < 0.001) as the extraction method compared to UE and SE. 

The explanation lies in the use of different solvents in the extractions. H_2_O, which was used in UE, is the most polar solvent. Following in polarity is EtOH, used in SE. CO_2_ in SFE is non-polar, and therefore, EtOH was added as a co-solvent, which increases its polarity and thus enables better solubility and isolation of more polar phytochemicals such as polyphenols. Additionally, grinding the AS before the extraction processes allowed the reduction of particle size and increased the surface area, which contributed to higher extract diffusion (and consequently higher *η*) during the extraction process. Tan and colleagues [24] recently performed aqueous UE and achieved a 15.13% yield. SE with EtOH and SFE with EtOH as co-solvent was performed by Páramos et al. [21]. The study revealed that among the solvents, EtOH provided the best yield with SE (8.2–10.3%), while in SFE, the addition of EtOH as a co-solvent allowed yields from 1.9 to 6.9%. The higher yields of SE are mainly due to the longer extraction time and the greater amount of the more polar solvent used, which otherwise speaks in favor of SFE, leading to lower energy and material consumptions. According of the reviewed literature, a small number of studies reported on the extraction of biologically active compounds from AS using SFE, and even fewer reported extraction yields. The before-mentioned research by Páramos et al. [21] studied the influence of temperature and pressure, as main operating variables, on extraction efficiency. The highest extraction yield (6.9%) was achieved at 250 bar and 80 °C. Recently, Restrepo-Serna et al. [25] reported on a biorefinery approach for valorization of AS through SFE. SFE was conducted at 80 °C and 250 bar, and the yield of AS extract amounted to 0.12 g/g of raw material. On the other hand, Wang et al. [26] performed SFE at 50 °C and 250 bar using only CO_2_ as solvent and achieved a yield of 1.61%. On that account, the choice of using EtOH as a co-solvent turned out to be favorable. Hereafter, to increase the efficiency of SFE itself, it would be necessary to optimize important variables (operating conditions) in the process. 

### 2.2. Presence of Phytochemicals in AS Samples

The AS extracts were subjected to a phytochemical screening using various well-known standard qualitative methods. The results are shown in Table 1. 

It is important to highlight that alkaloids, flavonoids, phenolic compounds, saponins, steroids, terpenoids, and quinones were present in all three types of extracts. The research showed the absence of anthraquinones, carbohydrates, cardiac glycosides, emodins, phlobatannins, and tannins. Anthocyanins and coumarins were present in the UE and SE extracts, while they were absent in the SFE extract, which can be justified by explaining the polarity of the solvents used in the extraction process. Anthocyanins are known as water-soluble flavonoids [27] and were not extracted by SFE most likely due to the lower polarity of scCO_2_ and EtOH solvent mixtures. Coumarins are well soluble in EtOH and slightly soluble in H_2_O, while the solubility of scCO_2_ depends on the variables in the extraction process itself (operating pressure and temperature) [28,29], and therefore, it is possible that they were not extracted under the applied operating conditions. 

No phytochemical screening on SFE avocado seed extracts was detected in the reviewed literature, which means that this study is the first to provide additional insight into a more modern, green, and unconventional technique for extracting phytochemicals from AS. On the other hand, quite a few studies [30,31,32,33] with a preliminary screening of phytochemicals in AS extracts obtained by conventional extractions have already been published, but the results themselves are difficult to compare due to the different extraction methods, conditions, and solvents used. For example, Rivai et al. [34] found the presence of tannins and the absence of flavonoids, terpenoids, saponins, and steroids in EtOH and H_2_O extracts of AS, which is inconsistent with results in the presented study. On the contrary, Oboh et al. [10] found the presence of alkaloids, saponins, and terpenoids and the absence of anthraquinones and phlobatannins in the aqueous extract, which is in line with the presented results. Here, it is necessary to emphasize, however, that the chemical profile, the content, and activities of biological compounds from AS may differ due to the influence of the variety, origin, season, maturity, growth, post-harvest, and environmental conditions [35]. 

### 2.3. Content of Total Phenols, Proanthocyanidins, and Total Proteins in AS Samples

**Figure 2:**
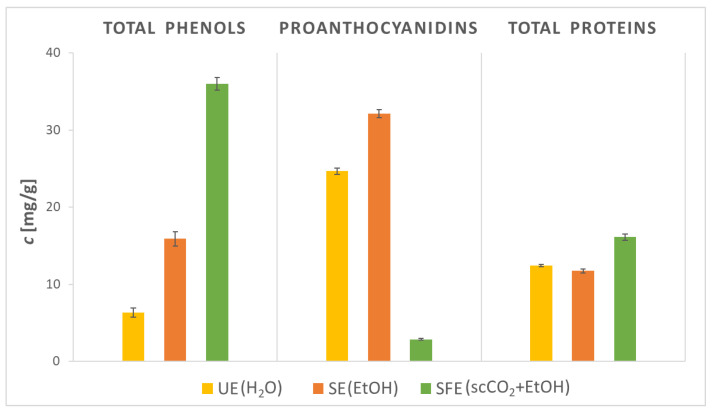
Content of total phenols (TPC), proanthocyanidins (PAC), and total proteins (PC) in AS extracts obtained by ultrasonic extraction (UE; solvent H_2_O), Soxhlet extraction (SE; solvent EtOH), and supercritical fluid extraction (SFE; solvent scCO_2_, co-solvent EtOH).

As seen in the previous subsection, the presence of phytochemicals in AS extracts, the method, and the solvent used greatly affected both the extraction efficiency and the successful extraction of various phytochemicals. Accordingly, a quantitative investigation of the content of TPC, PAC, and PC of the examined AS extracts was carried out. The results of the study are presented in Figure 2. 

TPC obtained with SFE was the highest among all extracts and amounted to 36.01 mg GAE/g of extract. Compared to SFE, TPC with UE (6.31 mg GAE/g) was almost six times lower, while TPC with SE (15.91 mg GAE/g) was almost two times lower than SFE. In the reviewed literature, the TPC values for AS extracts ranged up to 146.60 mg GAE/g with UE using hydroalcoholic mixtures as solvents [36], while no TPC values were detected in the reviewed literature using only H_2_O as a solvent. Segovia et al. [37] demonstrated that ultrasonic power and temperature have a huge impact on the extraction of polyphenols with H_2_O as solvent, as with the increase of the mentioned parameters, the TPC should also be enhanced. Therefore, it is likely that the UE efficiency of the present study is lower due to the low operating temperature (20 °C), and with more optimal conditions (higher operating temperature), the UE yield of AS extracts could be increased. With SE using EtOH as solvent, the TPC values ranged between 19.87–29.92 mg GAE/g [21,38], while with SFE, the TPC values reached up to 51.36 mg GAE/g [21]. Hence, the lower TPC value for SE compared to SFE is also justified, as SE needs higher operating temperatures to improve solvent solubility and to reduce its viscosity and surface tension, while high temperatures can lead to degradation of certain phenolic compounds and therefore lower TPC. On the other hand, low viscosity and near-zero surface tension allow scCO_2_ to easily penetrate the matrix material to extract phenolic compounds. Additionally, Kruskal–Wallis H test showed that there was a statistically significant difference between TPC prepared by different extraction methods. A post hoc Dunn–Bonferroni test revealed that there was statistically higher content of TPC present in extract obtained by SFE compared to SE and UE. 

Hereafter, PACs were determined in AS extracts. The experiments showed that the PAC value was the lowest in the SFE extract (2.84 mg/g), followed by the UE extraction (24.67 mg/g). The highest PAC value, 32.13 mg/g, was detected in the AS extract obtained with SE. After a thorough review of the already-published literature, it was found that the PAC value was not quantitatively determined comparatively in AS extracts. Only a study by Noorul et al. [39] determined PAC using the vanillin-H_2_SO_4_ protocol in AS extracts obtained by SE. The EtOH extract showed 13.70 μg catechin/mL and the H_2_O extract 9.30 μg catechin/mL. PACs are phytochemicals with added value, as their health aspects (e.g., antimicrobial, antioxidant, anticancer, antidiabetic, and neuroprotective activities) have been validated [40,41]. The mentioned results of the presented study are important data for potential further applications of AS extracts in biomedicine, pharmaceutical products, cosmetics, functional food, and nutraceuticals. Additionally, PC in AS extracts was examined. The highest PC value is contained in the SFE extract (16.10 mg/g), followed by the UE extract (12.43 mg/g) and finally the SE extract (11.74 mg/g). In the reviewed literature, quantitatively determined PC was not detected in AS extracts, but in some studies [42,43,44], proximate composition analyses were performed, where PC values reached up to 23.0%. The use of scCO_2_ affects the AS cells by damaging the cell walls, which results in the release of intracellular materials. Consequently, intracellular proteins contribute to PC [45], which explains the higher content in the SFE extract. Regarding content of PAC and PC, there was also a statistically significant difference using different extraction methods. 

### 2.4. Content of Certain Phenolic Compounds in AS Samples

A comprehensive analytical characterization of certain phenolic compounds was performed in AS extracts. The HPLC allowed effective and rapid separation and identification of 14 divergent phenolic compounds based on comparison of retention times with corresponding standards. The content of three flavonoids (epicatechin, hesperidin, and quercetin), ten phenolic acids (benzoic acid, 2,3-dihydroxy benzoic acid, 4-hydroxy benzoic acid, caffeic acid, chlorogenic acid, cinnamic acid, p-coumaric acid, ferulic acid, gallic acid, and salicylic acid) and vanillin was determined in UE, SE, and SFE extracts of AS, which are presented in Table 2.

Regarding the flavonoids in AS extracts, the common fact between the UE and SE extracts is that hesperidin predominated (118.10–226.78 mg/100 g DW), while its content was lower in the SFE extract (9.37 mg/100 g DW). In comparison, hesperidin was also detected by Zaki et al. [46], but its content was much higher in peel extracts (up to 61.94 mg/100 g DW) than in seed extracts (up to 2.62 mg/100 g DW). However, the presence of quercetin was found in the SFE extract (58.07 mg/100 g DW), while it was not detectable in the remaining extracts. In the reviewed literature, different quercetin contents in AS extracts are reported, ranging from 0.07 to 88.18 mg/100 g [5,19,46,47,48]. Since epicatechin is a building block of proanthocyanidins, whose total content was high, especially in the UE extract, it was unambiguously identified in all AS extracts (15.56–39.20 mg/100 g DW) by comparing the retention time with that of the standards. It has also been confirmed many times in similar AS extracts in the reviewed literature [5,16], up to a content of 2.91 mg/100 g DW.

When comparing the presence of valuable phenolic acids in AS extracts, 2,3-dihydroxybenzoic acid (also known as pyrocatechuic acid) content was the highest (14.42–106.48 mg/100 g DW) in all extracts (UE > SE > SFE). The same sequence of the AS extracts (UE > SE > SFE) can also be detected in the content of 4-hydroxybenzoic acid, which is also known as *p*-hydroxybenzoic acid (3.47–15.41 mg/100 g DW), while benzoic acids were found only in UE extract. All mentioned acids were also identified in the reviewed literature [5,47,49]. Caffeic acid has only been detected in SFE extract at a content of 5.92 mg/100 g DW, while chlorogenic acid appeared in all AS extracts (UE > SE > SFE, 0.73–9.36 mg/100 g DW) as well as cinnamic acid (UE > SFE > SE, 11.33–34.36 mg/100 g DW). The content of caffeic acid (4.18–60.19 mg/100 g), chlorogenic acid (0.05–2.61 mg/100 g), and cinnamic acid (up to 0.09 mg/100 g) was previously determined by other researchers [5,19,46,48]. Next, the highest content of p-coumaric acid was detected in the SE extract (6.26 mg/100 g DW), followed by the SFE and UE extracts. The literature indicates p-coumaric acid content in AS extracts from 0.62 to 10.23 mg/100 g [19,46,48]. Hereafter, ferulic acid was found in SE and UE extracts (up to 6.86 mg/100 g DW), gallic acid in SFE and UE extracts (up to 3.99 mg/100 g DW), and salicylic acid in SE and SFE extracts (up to 15.15 mg/100 g DW). For comparison, the content values for ferulic acid in the reviewed literature are between 0.09–82.53 mg/100 g, for gallic acid between 1.21–67.38 mg/100 g, and for salicylic acid between 2.57–5.50 mg/100 g [5,19,46,48]. Finally, vanillin was also previously determined in AS extracts [47] and is known as an antimicrobially active phenolic compound [50,51]. In the presented study, vanillin was identified in all extracts (UE > SE > SFE) and quantified up to a content of 55.02 mg/100 g DW. 

Overall, the highest total content of analyzed phenolic compounds was contained in UE extract (393.94 mg/100 g DW), followed by SE extract (351.03 mg/100 g DW) and SFE extract (177.99 mg/100 g DW). In the presented study, compared to the contents of other authors, the high contents of hesperidin, epicatechin, cinnamic acid, and salicylic acid stood out. Additionally, 2,3-dihydroxybenzoic acid, 4-hydroxybenzoic acid, benzoic acid, and vanillin have been identified in already-published research, while their quantification in AS extracts has not yet been detected. Importantly, many studies have examined the content of different phenolic compounds in AS extracts obtained by different extraction methods and under different conditions and with different solvents. Therefore, it must be emphasized that there are certain deviations in the results of various studies, as the chemical profile and the content of biologically active compounds in AS extracts differ due to the previously mentioned different extraction conditions as well as due to the variety and maturity of the fruit used in the studies. What gives great importance to the presented study is the characterization and quantification of selected flavonoids and phenolic acids in SFE extracts since no similar study could be detected in the reviewed published literature heretofore. However, the optimization of important variables (operating conditions) in SFE is also important here, which could further increase the efficiency of the recovery of phenolic compounds. In any case, for the extraction and potential isolation of an individual compound, it would be necessary to carefully study the influence of the operating conditions since the solubility trend changes for certain compounds [23].

### 2.5. Enzyme Activities of AS Samples

Since plants are a valuable source of enzymes [52], a comparative study of important enzyme activities in AS extracts was carried out. The obtained results of the study are delineated in Table 3.

The results of the study showed cellulase activity only in the SFE extract. Cellulases are extremely applicable enzymes in many industries and have recently shown good potential in the fight against antibiotic-resistant bacteria [53] and in the conversion of agricultural waste into bioethanol and sugar [54]. Furthermore, the highest lipase activity was demonstrated in the UE extract (56.30 U/g), followed by the activity in the SFE extract (24.54 U/g), while lipase activity was not detected in the SE AS extract. Since lipases catalyze the hydrolysis of ester-carboxylate bonds and release organic alcohols and fatty acids with high efficiency and stability [55], they are extremely appealing from a commercial point of view for quite a few industries. Many different seeds have already been demonstrated as a potential source for possible lipase exploitation [56], and with this study, AS have also become an attractive alternative. Thereafter, peroxidases are important antioxidant enzymes that are also applied in medicine, analytics, agriculture, and other fields [57]. Peroxidase was active in all AS extracts, but compared to other analyzed enzymes, its activity was the lowest. On the contrary, the polyphenol oxidase (PPO) activity was generally the highest and was expressed in all extracts (SE > UE > SFE, up to 4250.00 U/g). The results are not surprising since PPO is the enzyme responsible for the browning. In the presence of oxygen, PPO changes phenolic compounds into various quinones through the oxidation process, which further react and form melanin. Melanin is dark pigment that colors the fruits/seeds brown. When AS are crushed in the presence of air, they soon develop a red-orange color [58]. Hatzakis et al. [59] discovered that AS extract contains a pigment called perseorangin, which is a yellow-orange solid and is the result of a PPO-dependent reaction. All AS extracts also resulted in protease (SFE > UE > SE) and transglutaminase activity (UE > SE > SFE). Plant proteases are also actively used in medicine, as they exhibit a wide spectrum of therapeutic actions. Moreover, antimicrobial, antioxidant, anti-inflammatory, and antidiabetic properties of bioactive peptides obtained from plant proteases have also been proven [60]. Transglutaminase, in addition to other applications (e.g., food additives), may be considered as an innovative category of wound-healing mediators [61,62]. Next, superoxide dismutase (SOD) activity has been evaluated in AS extracts. Specifically, SE and UE AS extracts demonstrated high SOD activity (up to 3123.97 U/g), while the activity in SFE extract was slightly lower. Due to their antioxidant effects, SODs have enormous potential for applications in many industries, including medicine, as an abundant number of studies have reported their physiological importance and therapeutic potential [63]. Importantly, to the best of our knowledge, only studies containing enzyme-inhibitory potential against specific enzymes [64,65] are found in the reviewed literature, while no similar comparative study covering the activity of selected enzymes in any AS extracts has been published so far. Accordingly, the presented results greatly contribute to the identification of valuable enzymes in AS, which could potentially be a source for their exploitation and further applications in divergent branches and industries. However, it is important to point out that only the SFE extract contains all the tested enzymes in their active form; therefore, the SFE extract is the most suitable source of active enzymes from AS of all tested extracts.

### 2.6. Antioxidant Activity of AS Samples

**Figure 3:**
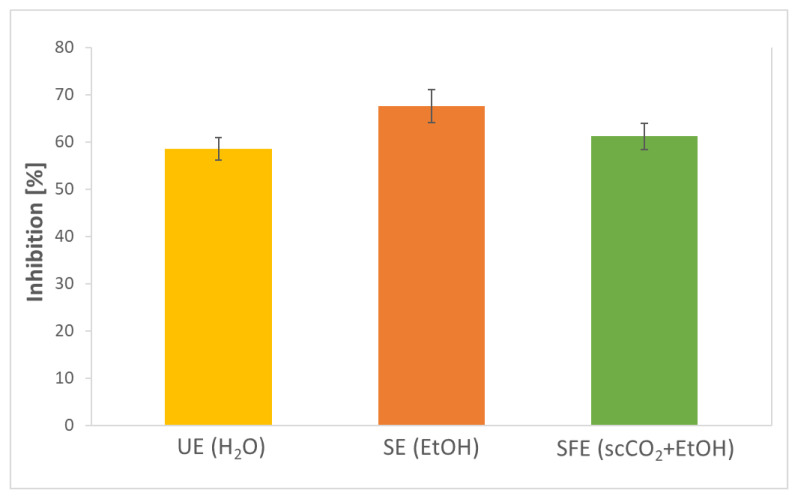
Antioxidant activities of AS extracts obtained by ultrasonic extraction (UE; solvent H_2_O), Soxhlet extraction (SE; solvent EtOH), and supercritical fluid extraction (SFE; solvent scCO_2_, co-solvent EtOH).

Plants contain a large number of bioactive compounds with high antioxidant activity. Studies of the antioxidant activity of various plant species contribute to revealing the value of these species as a source of new antioxidant compounds. Therefore, the antioxidant potential of AS extracts was examined. The results are depicted in Figure 3. 

Using the DPPH method, the extracts showed inhibition in the range of 58.50–67.49%. The highest antioxidant activity was detected in SE extract, followed by SFE and UE extract. Health-promoting biologically active compounds (e.g., phenolic compounds) definitely contribute to the antioxidant potential of the extracts. Various studies indicate that some phenolic compounds (epicatechin, quercetin, benzoic acid, caffeic acid, chlorogenic acid, ferulic acid, 4-hydrohibenzoic acid, and p-coumaric acid) also quantified in the presented study have already been proven to be associated with the antioxidant activity of AS extracts [66].

Nonetheless, the before-mentioned high enzyme activity of the SOD enzyme in AS extracts, which is well known for its antioxidative effects, should be emphasized. Kruskal–Wallis H test showed that there was a statistically significant difference in the antioxidant activity of AS extracts prepared by different extraction methods. For comparison, Weremfo et al. [19] detected 73.61% inhibition of AS EtOH extract obtained by microwave-assisted extraction (MAE) and 60.56% inhibition of extract obtained by conventional solvent extraction (CSE). In the present study, 67.49% inhibition was achieved with the same EtOH solvent, but the extraction method was different (SE). In comparison, the extraction method has an effect on the antioxidant potential of the extracts, as SE in the present study resulted in a higher *I* than CSE, while it demonstrated lower *I* than MAE. Furthermore, Zaki et al. [46] determined up to 97.84% inhibition (MeOH extracts), while Al-Juhaimi et al. [48] determined up to 83.70% inhibition using UE (hexane, MeOH/H_2_O extracts). The importance of the solvent used with the same extraction method can also be detected here, as with other solvents (MeOH, hexane, etc.) AS extracts showed a higher antioxidant potential than the presented UE H_2_O extract (58.50%). Recently, Kautsar et al. [67] studied the effect of AS extract storage on antioxidant activity, which decreased from 91.58 to 84.05% after 4 weeks. Differences between studies also arise as a result of the influence of many factors such as the variety, origin, and maturity of the fruit, and as mentioned, additional discrepancy is caused by the use of different methods and solvents for the extraction of biologically active compounds.

### 2.7. Antimicrobial Activity of AS Samples

The increase in antimicrobial resistance, the decrease in the effectiveness of synthetic drugs, and, at the same time, their increased toxicity has led to an ever-increasing search for alternative biologically active substances. As AS are a by-product of the avocado industry, they have already been investigated as a potential source of antimicrobial compounds. Data in previously published studies indicate that diverse AS extracts inhibit the growth of *Candida* spp., *Cryptococcus neoformans*, *Malassezia pachydermatis* [4], *Corynebacterium ulcerans*, *Escherichia coli*, *Staphylococcus aureus*, *Streptococcus pyogenes*, *Salmonella typhi* [30], *Entamoeba histolytica*, *Giardia lamblia*, *Trichomoniasis vaginalis*, *Mycobacterium tuberculosis* [68], *Clostridium sporogenes* [69], *Proteus mirabilis*, *Pseudomonas aeruginosa*, *Aspergillus niger* [70], *Porphyromonas gingivalis* [71], *Bacillus cereus*, *Listeria monocytogenes*, *Pseudomonas* spp., *Yarrowia lipolytica* [72], *Klebsiella pneumoniae* [3], *Staphylococcus epidermidis*, *Enterococcus faecalis*, *Salmonella enteritidis*, *Citrobacter freundii*, *Enterobacter aerogenes*, *Zygosaccharomyces bailii*, *Aspergillus flavus*, and *Penicillium* spp. [73]. Many studies have investigated the antimicrobial effect of AS extracts obtained by well-known conventional methods on plenty of microorganisms. On the other hand, it should be point out that so far, it is possible to find more than one a recent study, which is by David et al. [74], that tested the antimicrobial effect of AS extracts obtained with greener SFE. The susceptibility of *L. monocytogenes*, *S. typhimurium*, and *E. coli* to AS extracts obtained by scCO_2_ at different operating temperatures and pressures was studied. Extracts obtained at 40 °C and 30 MPa and at 50 °C and 20 MPa showed only inhibition of *L. monocytogenes* growth. Hence, the presented study is a major contribution to this field, as it includes a comprehensive and comparative study of the qualitative and quantitative antimicrobial efficacy of UE, SE, and SFE AS extracts on 15 different microorganisms.

Initially, the antimicrobial activity was tested qualitatively using the disc diffusion method. The results are shown in Table 4. 

Promisingly, all AS extracts inhibited the growth of all selected Gram-negative bacteria. The inhibition zone with the addition of AS extracts obtained with different methods on *P. aeruginosa* was the same, while the addition of SE extract to *E. coli* and *P. fluorescens* resulted in the largest inhibition zone. Furthermore, SE extract inhibited the growth of all Gram-positive bacteria, while *B. cereus* was not susceptible to the addition of UE extract and *S. pyogenes* to the addition of UE and SFE extracts. The sensitivity of fungi to AS extracts was also examined. The SE extract showed the lowest antifungal performance, as it only inhibited the growth of *P. cyclopium*. UE extract only inhibited the growth of *A. fumigatus* and *P. cyclopium*. On the contrary, the SFE AS extract was very effective as an antifungal agent, as it inhibited the growth of six out of eight selected fungi; only *S. cerevisiae* and *T. viride* were not susceptible to its addition. Overall, using the disc diffusion method, SFE AS extract proved to be the most antimicrobially effective, inhibiting the growth of 11 out of 15 microorganisms, followed by SE (8/15) and UE extract (7/15).

**Figure 4:**
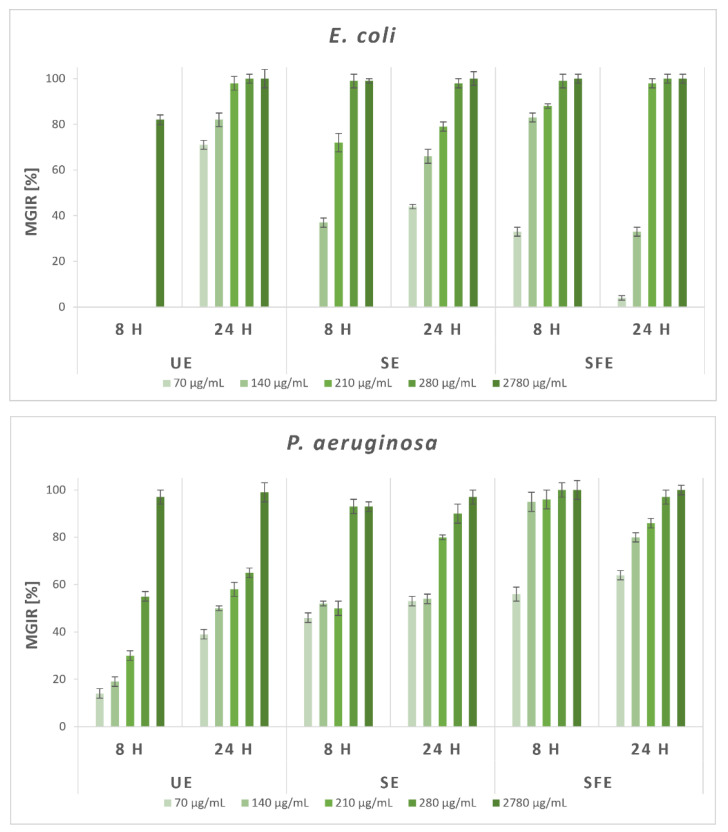

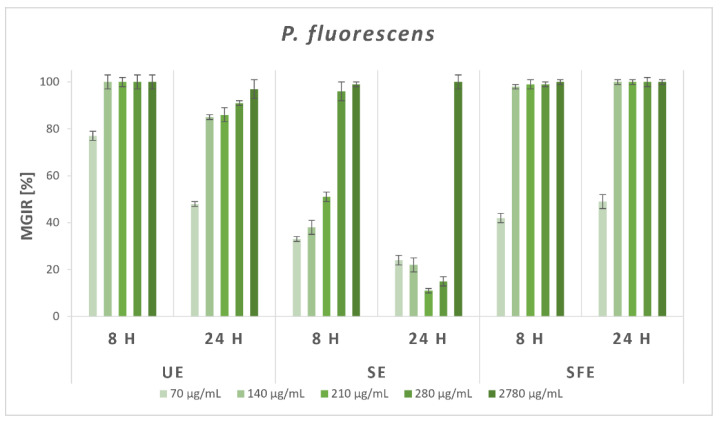
Microbial growth-inhibition rates (MGIRs) for AS extracts obtained by ultrasonic extraction (UE; solvent H_2_O), Soxhlet extraction (SE; solvent EtOH), and supercritical fluid extraction (SFE; solvent scCO_2_, co-solvent EtOH) using 70, 140, 210, 280, and 2780 μg sample per mL of selected Gram-negative bacteria suspension.

Given the promising results of the qualitative study, the antimicrobial effectiveness of AS extracts was quantified using broth microdilution method. The MGIRs were determined for selected Gram-negative and Gram-positive bacteria and fungi at five different concentrations of AS extracts. A comparison of MGIRs after 8 and 24 h was also carried out, which enables a more comprehensive insight of the antimicrobial activity of AS extracts for possible applications in various industries. According to all the reviewed literature, similar studies containing a certain percentage level of growth inhibition for selected microorganisms with any AS extracts have not yet been published. Therefore, Figure 4 shows the results of a quantitative study of the antimicrobial efficacy of AS extracts against selected Gram-negative bacteria, which was carried out by broth microdilution method.

After 24 h of incubation with the highest added concentration of inhibitors, all AS extracts completely inhibited growth of Gram-negative *E. coli* (with 100% MGIR). More specifically, UE extract at the concentration of 2780 μg/mL inhibited MGIR by 82% after 8 h of incubation, while *E. coli* was then not susceptible to lower concentrations. After 24 h, the addition of the lowest UE extract concentration (70 μg/mL) resulted in 71% MGIR and addition of 210 μg/mL in as much as 98% MGIR. Furthermore, the addition of SE extract at a concentration of 210 μg/mL showed 72% MGIR after 8 h, and after 24 h, more than 50% inhibition of *E. coli* growth was achieved with 140 μg/mL. At both times, almost complete inhibition (99 and 98% MGIR) was achieved with the addition of 280 μg/mL SE extract as an inhibitor. The SFE extract also proved to be a good inhibitor of the growth of *E. coli*, which already showed 33% MGIR after 8 h with the concentration of 70 μg/mL as the lowest concentration and as much as 83% MGIR with 140 μg/mL. After 24 h, the concentration of 210 μg/mL SFE extract reached as much as 98% MGIR.

Gram-negative *P. aeruginosa* was the least susceptible to the addition of UE extract, as only the highest added concentration of the extract achieved its complete growth inhibition. Lower concentrations of UE extract, however, resulted in MGIRs of up to 65%. On the contrary, SE and SFE extracts even with the addition of the lowest concentration, 70 μg/mL, showed MGIRs between 46–64% after 8 and 24 h. In the case of the addition of SE extract as an inhibitor, higher-than-90% MGIRs were achieved with a concentration of 280 μg/mL. However, the SFE extract proved to be exceptionally effective as a growth inhibitor of *P. aeruginosa*, as it reached 95% MGIR after 8 h of incubation with 140 μg/mL of the added extract and 97% MGIR after 24 h of incubation with 280 μg of SFE extract per mL of suspension. 

Exceptional results of antibacterial efficiency were achieved with the addition of UE and SFE extracts as inhibitors on the growth of Gram-negative *P. fluorescens*. After 8 h of incubation, the addition of 70 μg/mL UE extract resulted in 77% MGIR, while 140 μg/mL and higher concentrations showed complete inhibition of *P. aeruginosa* growth. After 24 h of incubation, the lowest concentration inhibited its growth with 50% MGIR, and further concentrations resulted in more than 85% MGIRs. After 8 h of incubation of *P. aeruginosa* with 210 μg/mL SE extract, the result was 50% MGIR and 96% with the addition of 280 μg/mL. The mentioned bacterium was less susceptible to lower concentrations of SE extract after 24 h of incubation, but the highest concentration of 2780 μg/mL still completely inhibited its growth. Importantly, even the lowest added concentrations of SFE extract significantly affected the growth of *P. aeruginosa* (42 and 49% MGIR after 8 and 24 h), while the addition of 140 μg/mL SFE extract completely inhibited its growth in both time periods.

**Figure 5:**
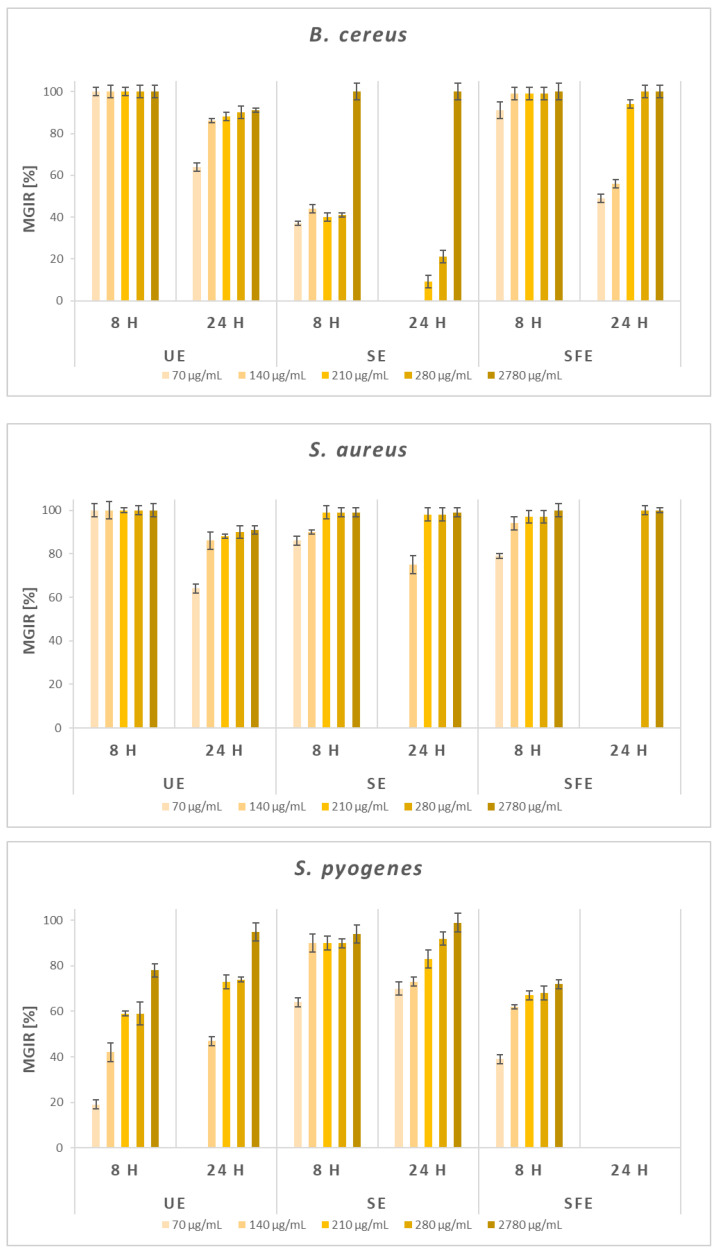
Microbial growth-inhibition rates (MGIRs) for AS extracts obtained by ultrasonic extraction (UE; solvent H_2_O), Soxhlet extraction (SE; solvent EtOH), and supercritical fluid extraction (SFE; solvent scCO_2_, co-solvent EtOH) using 70, 140, 210, 280, and 2780 μg sample per mL of selected Gram-positive bacteria suspension.

Figure 5 shows the results of a quantitative study of the antimicrobial efficacy of AS extracts against selected Gram-positive bacteria, which was carried out by BMM. 

Regarding Gram-positive *B. cereus*, generally higher antibacterial efficiency of AS extracts was observed after 8 h of incubation, while after 24 h, the efficiency decreased except at the highest added concentration. The UE extract has a remarkable impact on the *B. cereus*, as it completely inhibited (100% MGIR) its growth even at the lowest concentration after 8 h of incubation. The results were similar regarding SFE extract, starting with 91% MGIR at 70 μg/mL. *B. cereus* was least susceptible to the addition of SE extract. After 8 h of incubation, concentrations of SE extract in the range of 70–280 μg/mL resulted in 37–41% MGIRs, while 2780 μg/mL completely inhibited the growth of *B. cereus*.

AS extracts were shown to be good growth inhibitors of Gram-positive *S. aureus*. Similar to *B. cereus*, generally, after 8 h of incubation even the lowest added concentrations, AS extracts greatly inhibited the growth of *S. aureus* (79–100% MGIRs). Again, all tested concentrations of UE extract completely inhibited the growth of the mentioned Gram-positive bacteria after 8 h, and after 24 h, the MGIRs increased from 64 to 91%. Moreover, 70 μg/mL of SE extract resulted in 86% MGIR after 8 h, and further concentrations inhibited the growth of *S. aureus* with 90 and 99% MGIRs. After 24 h, 210 μg/mL of SE extract completely inhibited its growth. Finally, SFE extract inhibited *S. aureus* growth with 79% MGIR (8 h) when added at 70 μg/mL, and MGIRs increased with a concentration up to 100%. *S. aureus* was not susceptible to the three lowest concentrations of SFE extract after 24 h of incubation, although 280 and 2780 μg/mL completely inhibited its growth.

The effect of the addition of AS extracts as inhibitors on Gram-positive *S. pyogenes* was also investigated. In contrast to *B. cereus* and *S. aureus*, the greatest antibacterial potential was shown by SE extract, where even lower concentrations resulted in higher MGIRs compared to UE and SFE extracts. After 8 h, 140 μg/mL of SE extract inhibited the growth of *S. pyogenes* by 90% and after 24 h by 73%, while higher concentrations resulted in MGIRs ranging between 83 and 99%. UE extract showed a higher level of inhibition after 24 h (47–95% MGIRs). In contrast, SFE extract showed a certain level of inhibition (39–72% MGIRs) after 8 h of incubation, while *S. pyogenes* was not susceptible to the addition of SFE extract after 24 h of incubation, which is in agreement with the findings of the disc diffusion method.

Using the broth microdilution method, it could be estimated that, in general, when lower concentrations of AS extracts were added, Gram-positive bacteria were more sensitive and susceptible to the addition of AS extracts, which is in line with the claims of other authors [72]. This can be explained by the fact that Gram-negative bacteria are more resistant to the addition of inhibitors due to the presence of an additional protective outer membrane, which Gram-positive bacteria lack [75].

**Figure 6:**
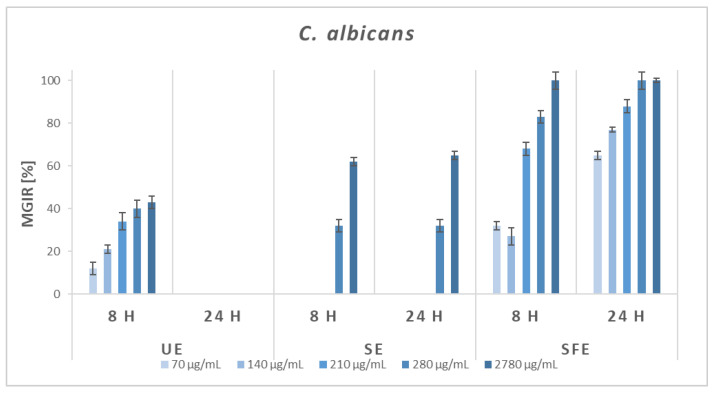
Microbial growth-inhibition rates (MGIRs) for AS extracts obtained by ultrasonic extraction (UE; solvent H_2_O), Soxhlet extraction (SE; solvent EtOH), and supercritical fluid extraction (SFE; solvent scCO_2_, co-solvent EtOH) using 70, 140, 210, 280, and 2780 μg sample per mL of *C. albicans* suspension.

Furthermore, the antifungal activity of AS extracts was also investigated. Since most fungi form spores, which makes them incompatible with the broth microdilution method, the quantitative antifungal efficacy of UE, SE, and SFE extracts was tested against *C. albicans*. The results are shown in Figure 6. 

The results are in accordance with the disc diffusion method because even when using BMM, only the SFE extract showed good antifungal efficiency. UE extract resulted in 12–43% MGIRs after 8 h of incubation, but after 24 h, the fungus was not sensitive to the addition of the extract at all. In the case of the addition of SE extract as an inhibitor, lower concentrations did not affect the growth of *C. albicans*, and the two highest concentrations (2780 and 280 μg/mL) reached 32–65% MGIRs. On the other hand, the SFE extract was more effective against *C. albicans*. A higher degree of growth inhibition was achieved after 24 h of incubation, when even 70 μg/mL of SFE extract showed 65% MGIR, and complete inhibition was achieved with the addition of 210 μg/mL.

Antimicrobial activity is attributed to many phytochemicals or biologically active compounds. Other authors [76,77,78] mainly cite the antimicrobial effect of phenolic compounds, which change the function of bacterial cell membranes and thereby slow down growth and inhibit bacterial reproduction. Furthermore, the antimicrobial effect of fatty acids (e.g., palmitic acid) and their derivatives acetogenins (e.g., avocadene, persin, persediene, and persenone A, B, and C) obtained from AS is also reported [79,80]. The fluidity, disorganization, and also the disintegration of the cell membranes occur due to disordering of the phospholipid chain, which is the cause of the leakage of intracellular content and, consequently, cell death [81]. However, the correlation between the identified phenolic compounds in the obtained AS extracts and their antimicrobial effectiveness is important. The antibacterial/antifungal activity of hesperidin [82], quercetin [83], benzoic acid [84], 2,3-dihydroxybenzoic acid [85], 4-hydroxybenzoic acid [86], caffeic acid [87], chlorogenic acid [88], cinnamic acid [89], p-coumaric acid [90], ferulic and gallic acid [91], salicylic acid [92], and o-vanillin [50] has already been demonstrated. It is possible to conclude that the high content of the mentioned phenolic compounds synergistically affects the antimicrobial efficiency of the obtained AS extracts.

For ease of review, MIC_90_ values were also determined from the BMM results as the concentrations at which AS extracts inhibited the growth of a particular bacterium/fungus by at least 90% of the MGIR. The results are shown in Table 5. 

Only a small number of studies covering MIC values for AS extracts can be detected in the literature. Nwaoguikpe and colleagues [3] determined MIC values for *E. coli*, *P. aeruginosa*, and *S. aureus* in the range of 40,000–50,000 μg/mL for aqueous, methanolic, and ethanolic AS extracts. Furthermore, a study by Idris et al. [30] resulted in MIC values for petroleum ether, chloroform, ethyl acetate, and methanol SE AS extracts in the range of 10,000–50,000 μg/mL for *E. coli*, *P. aeruginosa*, *S. aureus*, *S. pyogenes*, and *C. albicans*. Raymond et al. [73] determined MIC values for *P. aeruginosa* of 250.0 ± 216.5 μg/mL and for *E. coli* and *S. aureus* of greater than 500 μg/mL with ethanolic AS extract (Hass variety). MIC values for Gram-negative *P. fluorescens* and Gram-positive *B. cereus* have not been studied in the literature so far. In the presented research, the lowest MIC value was determined for *B. cereus* after 8 h of incubation in the case of UE and SFE extracts (70 μg/mL) compared to all tested microorganisms. MIC values were also determined for *P. fluorescens* and were especially promising for SFE extract (140 μg/mL) after 8 and 24 h of incubation. Compared to the previously listed research, our extracts showed incomparably lower MIC values for the remaining microorganisms, which greatly contributes to the current information on the antimicrobial activity of AS extracts.

For further applications in which it is necessary to consider, for example, the release of AS extract as a potential antimicrobial agent and the MIC for microorganisms after a certain incubation, contact time is extremely important. In the presented study, it was demonstrated that microorganisms are differently susceptible to the addition of diverse AS extracts and are variously sensitive to the addition of inhibitors after certain periods of time. For example, *S. aureus* is more susceptible to the addition of all three studied AS extracts after 8 h (lower MIC_90_ values) than after 24 h incubation time. In general, SFE extract compared to UE and SE extract resulted in lower MIC_90_ values, which is a great contribution to research in the field of the antimicrobial action of SFE AS extracts. 

## 3. Materials and Methods

### 3.1. Chemicals, Reagents, and Microorganisms

Chemicals including malt extract, potato dextrose broth, Triton X-100, tryptic soy broth, and tryptone were obtained from Fluka, Buchs, Switzerland. Mueller–Hinton broth (MHB) and potato dextrose agar (PDA) were purchased from Biolife, Milano, Italy. 4-Aminoantipyrine (4-APP), Coomassie Blue G-250, ethanol (EtOH, ≥99.5%), ferric chloride (FeCl_3_), hydrochloric acid (HCl, 37.0%), iodine (I_2_), meat extract, meat peptone, n-butanol (≥99.5%), 1-napthol (≥99.0%), phosphoric acid (85.0%), potassium dihydrogen phosphate (KH_2_PO_4_), potassium iodide (KI), sodium chloride (NaCl), sodium dihydrogen phosphate monohydrate (NaH_2_PO_4_·H_2_O), and sodium hydrogen phosphate (Na_2_HPO_4_) were from Merck, Darmstadt, Germany. Calcium chloride (CaCl_2_), D-(+)-glucose anhydrous, and ferrous sulfate heptahydrate (Fe(SO_4_)·7H_2_O) were purchased from Kemika, Zagreb, Croatia. Carbobenzoxy-L-Glutaminylglycine (CBZ-Gln-Gly) was obtained from Zedira GmbH, Darmstadt, Germany. Acetic acid (glacial, ≥99.7%), acetonitrile (≥99.9%), agar, ammonium hydroxide solution (NH_4_OH), ascorbic acid, benzoic acid (≥99.5%), bovine serum albumin (BSA), caffeic acid (98.0%), casein, chloroform (CHCl_3_, ≥99.0%), chlorogenic acid (≥95.0%), cinnamic acid (≥99.0%), 2,3-dihydroxybenzoic acid (99.0%), L-3,4-dihydroxyphenylalanine (L-DOPA, ≥98.0%), 3,5-dinitrosalicylic acid (DNS), 2,2-diphenyl-1-picrylhydrazyl (DPPH, ≥97.0%), ellagic acid (≥95.0%), (-)-epicatechin (≥90.0%), ethylenediaminetetraacetic acid (EDTA, 98.5–101.5%) ferulic acid (99.0%), Folin–Ciocalteu’s phenol reagent (FC), gallic acid (GA, ≥97.5%), glucose assay, hesperidin (≥97.0%), hydrogen peroxide (H_2_O_2_), hydroxylamine hydrochloride (HONH_2_·HCl), L-glutamic acid (99.0%), L-glutathione reduced (≥98.0%), maltose, methanol (MeOH, ≥99.9%), o-vanillin (99.0%), yeast extract, peptone from soybean, phenol (C_6_H_5_OH), p-coumaric acid (98.0%), p-hydroxy benzoic acid (99.0%), p-nitrophenyl butyrate (p-NPB, ≥98.0%), potassium sodium tartrate tetrahydrate (KNaC_4_H_4_O_6_·4H_2_O, 99.0%), 2-propanol (99.9%), pyrogallol (≥99.0%), quercetin (≥95.0%), salicylic acid (≥99.0%), Sigmacell cellulose, sodium acetate (CH_3_COONa, ≥99.0%), sodium carbonate (Na₂CO₃, ≥99.5%), sodium hydroxide (NaOH, ≥95.0%), starch, sulfuric acid (concentrated, H_2_SO_4_) trichloroacetic acid (TCA, ≥99.0%), and Trizma Base (NH_2_C(CH_2_OH)_3_, ≥99.7%) were purchased from Sigma-Aldrich, St. Louis, USA. Carbon dioxide (CO_2_, purity 2.5) was obtained from Messer, Ruše, Slovenia. 

Selected microorganisms including bacteria (*B. cereus* (DSM 345), *E. coli* (DSM 498), *P. aeruginosa* (DSM 1128), *P. fluorescens* (DSM 289), *S. aureus* (DSM 346), *S. platensis* (DSM 40041), and *S. pyogenes* (DSM 11728)) and fungi (*A. brasiliensis* (DSM 1988), *A. flavus* (DSM 818), *A. fumigatus* (DSM 819), *A. niger* (DSM 821), *C. albicans* (DSM 1386), and *S. cerevisiae* (DSM 1848)) were purchased from DSMZ-German Collection of Microorganisms and Cell Cultures GmbH from Berlin, Germany. The fungi *P. cyclopium* and *T. viride* were gifted from the Department of Agricultural Chemical Technology, Budapest University of Technology and Economics, Hungary.

### 3.2. Plant Material and Preparation of Samples

Avocado fruits (Hass variety) were stored at room temperature until full ripeness. Complete avocado seeds were then manually separated from the ripe avocado fruits and washed under the continuous flow of tap water. Obtained avocado seeds (AS) were then chopped into small pieces in order to accelerate the drying process. The final drying process took place at room temperature to avoid any effect of higher temperature on the content of bioactive compounds. Sliced, dried seeds (including seed coats) were then evenly ground and stored at room temperature and protected from light until their extraction and further analysis.

Extractions of dried AS were performed using different extraction methods in order to compare the content of various phytochemicals and bioactive compounds and to evaluate their bioactivity. First, under UE, the mixture of 20 g of dried AS and 150 mL of water (H_2_O) as solvent was exposed to ultrasonic irradiation at 40 kHz for 3 h at 20 °C using an ultrasonic bath (Iskra PIO-Sonis 4, Iskra, Šentjernej, Slovenia). After extraction, the mixture was filtered using Büchner funnel and flask to remove solid particles. Furthermore, for SE, about 25 g of dried AS was added into porous cellulose thimble and placed in Soxhlet extractor. A volume of 150 mL EtOH as a solvent was added into flask, attached to extractor and condenser, and heated to reflux. Extractions were conducted for 6 h. SFE of dried AS was performed using a semi-continuous apparatus [93]. To achieve better extraction of phenolic compound, EtOH was added as a co-solvent. Approximately 20 g of dried AS was placed into the extractor (60 mL), which was placed into a water bath preheated to operating temperature (40 °C). SFEs were performed at an operating pressure of 200 bar. The flow rate of CO_2_ was maintained at a constant of 2 mL/min while EtOH was pumped continuously using a high-pressure pump with a flow rate of 0.5–0.8 mL/min. The extractions took place for 2 h. Samples were collected in the previously weighted glass tubes at ambient conditions. After the conducted extractions, the solvents (H_2_O and EtOH) and co-solvents (EtOH) were evaporated under reduced pressure at 40 °C using the rotary evaporator (Büchi Rotavapor R-114, Flawil, Switzerland). The final extracts were stored and kept in a freezer at −20 °C until further use. 

### 3.3. Process Efficiency Evaluation 

The extraction yields (*η*) are presented as mean values of two conducted extractions and were calculated as recently described by Kupnik et al. [23] using the equation below.

$$\eta \;\left[\%\right]=\left(\frac{m_{extract}}{m_{dry\;material}}\right)\times 100$$

where

η is extraction yield (%),$\;m_{extract\;}$is mass of extract (g), and $m_{dry\;material\;}$is mass of dry AS (g).

### 3.4. Qualitative Determination of Phytochemicals

Phytochemical screening of extracts was conducted in order to qualitatively determine the presence of various phytochemicals and bioactive compounds. Prior to analysis, the extracts were prepared at a concentration of 1 mg/mL. Phytochemicals were determined using standard qualitative tests previously described in the literature [94,95,96], with slight modifications. The procedures and observations indicating a positive test are presented in Table 6. All experiments were carried out in triplicates, and results are presented based on visual observations.

### 3.5. Total Phenolic Content (TPC) Determination

The colorimetric method with Folin–Ciocalteu’s phenol reagent, accurately described by Leitgeb et al. [97], was used for determination of TPC. The results are reported as mg of gallic acid equivalents (GAE) per g of extract. Experiments were carried out in triplicates, and results are presented as mean value ± standard deviation (SD). 

### 3.6. Total Proanthocyanidins Content (PAC) Determination

The calorimetric method, where acid hydrolysis occurs using Fe(SO_4_)·7H_2_O in a mixture of HCl and *n*-butanol, accurately described by Leitgeb et al. [97], was availed for determination of PAC. The results are reported as mg of PAC per g of extract. Experiments were carried out in triplicates, and results are presented as mean value ± SD.

### 3.7. Total Protein Content (PC) Determination

PC was determined by Bradford method [98] using BSA as a standard. The results are reported as mg of total proteins per g of extract. Experiments were carried out in triplicates, and results are presented as mean value ± SD.

### 3.8. Identification and Quantification of the Phenolic Compounds

Prior to analysis, the extracts were prepared at a concentration of 10 mg/mL and filtered using 0.2 μm pore-size cellulose syringe filters. Extracts of dried AS were analyzed using an Agilent 1200 Series HPLC System equipped with a quaternary HPLC high-pressure pump, automatic sampler, column compartment, and variable wavelength detector (VWD). The chromatographic separation was performed with a Zorbax SB-C18 column (4.6 × 150 mm i.d., 5.0 μm particle size) at a flow rate of 2.0 mL/min using an injection volume of 10 μL. The column temperature was maintained at 25 ± 1 °C. The elution gradient consisted of acidified water (0.1% acetic acid, *v*/*v*) and acidified acetonitrile (0.1% acetic acid, *v*/*v*) as mobile phases A and B, respectively. To achieve efficient separation, the following multistep gradient program was used: 5% B (5 min), 10% B (10 min), 11% B (12 min), 18% B (18 min), 42% B (19 min), 50% B (24 min), 60% B (25 min), 70% B (28 min), and 5% B (30 min). Each standard and sample were analyzed in triplicate. The peaks were detected at 280 nm. In order to identify phenolic compounds in the extracts, their retention times were compared with corresponding standards. Additionally, all identified phenolic compounds were quantified using calibration curves. The results are reported as mg of certain phenolic compound per 100 g of DW. All experiments were carried out in triplicates, and results are presented as mean value ± SD.

### 3.9. Determination of Enzymatic Activity 

Specific spectrometric assays (Varian—CARY^®^ 50 UV–VIS Spectrophotometer, Varian Inc., The Netherlands) were availed for determination of activities of selected enzymes. Exact procedures for the determination of α-amylase, cellulase, lipase, peroxidase, protease, and transglutaminase activities are precisely described by Leitgeb et al. [97]. Polyphenol oxidase activity was determined using Creative Enzymes^®^ protocol [99], where the concentration of o-benzoquinone formed from L-DOPA by polyphenol oxidase was measured at 265 nm. Superoxide dismutase activity was determined using a reaction of pyrogallol autoxidation at 325 nm, described by the Creative Enzymes^®^ protocol [100]. The results are reported as units (U) per g of extract. All experiments were carried out in triplicates, and results are presented as mean value ± SD.

### 3.10. Determination of Antioxidant Activity

DPPH free radical method, based on electronic transfer that produces a purple-colored solution in EtOH and accurately described by Leitgeb et al. [97], was used for determination of antioxidant activity. The results are reported as a percentage of inhibition (*I*) relative to reference solution. Experiments were carried out in triplicates, and results are presented as mean value ± SD.

### 3.11. Determination of Antimicrobial Activity

First, the disc diffusion method, previously described by Kupnik et al. [101,102], was used to qualitatively assess the antimicrobial activity of the analyzed extracts. Tests were carried out at selected concentrations of microorganisms (1–5 × 10^6^ CFU/mL). Deionized H_2_O and 5% DMSO were used as negative controls, while 30 μg of amoxicillin, nystatin, or vancomycin per disc was used as positive control. The results are reported as mm of inhibition zone. All experiments were carried out in triplicates, and results are presented as mean value ± SD.

Furthermore, the broth microdilution method [101,102] was used to quantitatively assess the antimicrobial activity of the analyzed extracts. Tests were carried out at selected concentrations of microorganisms (1–5 × 10^6^ CFU/mL) using MHB as a universal nutrient medium for all microbial strains. As a negative control, 5% DMSO was used. MGIRs were determined after 8 and 24 h of incubation under optimal conditions for the growth of each microbial strain for five concentrations of added extract (2780, 280, 210, 140, and 70 μg extract/mL of suspension). Additionally, the concentration at which the extract inhibited the growth of the microorganism with at least 90% MGIR was defined as MIC_90_. All experiments were carried out in triplicates, and results are presented as mean value ± SD.

### 3.12. Statistical Analysis

All statistical data analyses were performed using IBM^®^ SPSS^®^ Statistics. Statistical data analysis was performed to study differences between extraction methods. The normality of the distribution of data was tested using Shapiro–Wilk’s test. The homogeneity of variances was determine using Levene’s test. Differences between extractions were determined with one-way analysis of variances (ANOVA) followed by Tukey’s post hoc test (for normally distributed data) and with nonparametric Kruskal–Wallis H test, followed by pairwise comparison using the Dunn–Bonferroni post hoc method (for abnormally distributed data).

## 4. Conclusions

Humanity is increasingly striving for a broader strategy for the shift and transition from a linear to a circular economy, including through a number of initiatives to reduce the inexhaustible waste generated annually. Food wastes present a renewable resource that can be collected and subsequently converted into value-added products, while reducing the volume of waste gathered in landfills and at the same time expanding the economic market share of new sustainable products. With this aim, avocado seeds were investigated as a potential source of biologically active compounds in extracts obtained by different methods. It was found that different solvents and extraction techniques affect both the extraction yield itself and the content of the selected biologically active compounds. Due to the different content of biologically active compounds, the enzymatic, antioxidant, and antimicrobial activities of AS extracts also differ. Importantly, compared to already known data, an extremely high content of hesperidin was found in SE (226.78 mg/100 g DW) and UE (118.10 mg/100 g DW) extracts. For the first time, the high content of 2,3-dihydroxybenzoic acid in the UE extract (106.48 mg/100 g DW) and vanillin (up to 55.02 mg/100 g DW) in all AS extracts was quantified. All three phenolic compounds are well known for their antimicrobial effects, so they most likely contributed to the good antimicrobial potential of the tested AS extracts. Furthermore, the high activity of the well-known antioxidant enzyme superoxide dismutase (up to 3123.97 U/g extract) most likely contributed to the antioxidant potential of AS extracts. However, AS extracts had a significant effect on various microorganisms, as they inhibited as many as 13 out of 15 tested fungi and bacteria. Only the fungi *S. cerevisiae* and *T. viride* were not susceptible to the addition of any of the AS extracts. Additionally, compared to the literature, extremely low MIC_90_ values were determined, starting with the lowest of 70 μg/mL for UE and SFE extract against the Gram-positive bacterium *B. cereus*. For this reason, the obtained AS extracts could be good alternative to synthetic antimicrobial agents. Overall, it is necessary to accentuate the AS extract obtained with a sustainable, greener, and modern SFE. The comprehensive study of the SFE extract in the presented research, which offers a comparison with the more well-known and conventional UE and SE, provides a great deal of new information. The SFE extract resulted in the highest content of total phenols and total proteins. It contained 12 of the 14 selected phenolic compounds and was the only one to show the activity of all the studied enzymes. It also emerged as the most promising antimicrobial agent, as it preliminarily inhibited as many as 11 out of 15 selected microorganisms and generally showed the lowest MIC_90_ values for 6 out of 7 investigated microorganisms.

Despite many published studies involving the phytochemistry and bioactivity of avocado seed extracts, the presented study provides additional knowledge and the contribution of important new information. The significant results of SFE provide additional insight into the extraction of bioactive ingredients from AS, while the results in the field of enzymatic activity and antimicrobial efficiency of AS extracts prove their extraordinary potential for further applications. Therefore, it would definitely make sense to exploit AS as a food waste for the recovery and production of value-added biologically active compounds, which could be further utilized in biomedicine, pharmacy, cosmetics, or other industries.

## Figures and Tables

**Table 1:** Screening for various phytochemicals in AS extracts obtained by ultrasonic extraction (UE; solvent H_2_O), Soxhlet extraction (SE; solvent EtOH), and supercritical fluid extraction (SFE; solvent scCO_2_, co-solvent EtOH).

Phytochemicals	UE (H_2_O)	SE (EtOH)	SFE (scCO_2_ + EtOH)
Alkaloids	+	+	+
Anthocyanins	++	+	-
Anthraquinones	-	-	-
Carbohydrates	-	-	-
Cardiac glycosides	-	-	-
Coumarins	+	++	-
Emodins	-	-	-
Flavonoids	+	+	+
Phenolic compounds	+	+	+
Phlobatannins	-	-	-
Saponins	+	+++	++
Steroids	++	++	+
Tannins	-	-	-
Terpenoids	+	+	++
Quinones	++	+++	+

The presence of phytochemicals is denoted by + (low amount), ++ (moderate amount), and +++ (abundant amount) and the absence by -.

**Table 2:** Content of certain phenolic compounds in AS extracts obtained by HPLC after ultrasonic extraction (UE; solvent H_2_O), Soxhlet extraction (SE; solvent EtOH), and supercritical fluid extraction (SFE; solvent scCO_2_, co-solvent EtOH).

		Content (mg_analyte_/100 g_DW_ ± SD)		
		UE (H_2_O)	SE (EtOH)	SFE (scCO_2_ + EtOH)
Flavonoids	(-)-Epicatechin	39.20 ± 0.68 ^a^	15.56 ± 0.02 ^b^	18.26 ± 0.06 ^b^
	Hesperidin	118.10 ± 1.51 ^a^	226.78 ± 2.39 ^a^	9.37 ± 0.85 ^b^
	Quercetin	-	-	58.07 ± 1.72
Phenolic acids	Benzoic acid	4.73 ± 0.17	-	-
	2,3-dihydroxybenzoic acid	106.48 ± 1.80 ^a^	34.26 ± 0.27 ^b^	14.42 ± 0.14 ^c^
	4-hydroxybenzoic acid	15.41 ± 0.19 ^a^	7.74 ± 0.10 ^b^	3.47 ± 0.11 ^b^
	Caffeic acid	-	-	5.92 ± 0.05
	Chlorogenic acid	9.36 ± 0.81 ^a^	2.24 ± 0.55 ^b^	0.73 ± 0.12 ^b^
	Cinnamic acid	34.36 ± 0.65 ^a^	11.33 ± 0.22 ^b^	13.06 ± 0.43 ^b^
	p-Coumaric acid	3.23 ± 0.05 ^a^	6.26 ± 0.06 ^b^	3.25 ± 0.06 ^a^
	Ferulic acid	4.97 ± 0.11 ^a^	6.86 ± 0.26 ^a^	-
	Gallic acid	3.08 ± 0.35 ^a^	-	3.99 ± 0.20 ^a^
	Salicylic acid	-	15.15 ± 0.56 ^a^	1.56 ± 0.18 ^b^
Other	o-Vanillin	55.02 ± 1.31 ^a^	24.85 ± 1.11 ^a^	45.89 ± 2.78 ^a^
Total content of analyzed phenolic compounds		393.94 ± 7.63	351.03 ± 5.54	177.99 ± 6.70

Each value represents mean of three replicates ± SD. Different letters in the same row indicate significant difference (*p* < 0.05).

**Table 3:** Enzyme activities of AS extracts obtained by ultrasonic extraction (UE; solvent H_2_O), Soxhlet extraction (SE; solvent EtOH), and supercritical fluid extraction (SFE; solvent scCO_2_, co-solvent EtOH).

Enzymes	Activity (U/g ± SD)		
	UE (H_2_O)	SE (EtOH)	SFE (scCO_2_ + EtOH)
Cellulase	-	-	30.74 ± 0.11
Lipase	56.30 ± 0.49 ^a^	-	24.54 ± 0.20 ^a^
Peroxidase	0.01 ± 0.00 ^a^	0.01 ± 0.00 ^a^	0.01 ± 0.00 ^a^
Polyphenol oxidase	4087.50 ± 71.43 ^a^	4250.00 ± 84.01 ^a^	3451.99 ± 47.17 ^b^
Protease	0.10 ± 0.01 ^a^	0.01 ± 0.00 ^a^	0.33 ± 0.03 ^b^
Transglutaminase	0.06 ± 0.01 ^a^	0.05 ± 0.00 ^a^	0.02 ± 0.00 ^b^
Superoxide dismutase	1435.97 ± 21.41 ^a^	3123.97 ± 69.05 ^b^	638.55 ± 15.99 ^c^

Each value represents mean of three replicates ± SD. Different letters in the same row indicate significant difference (*p* < 0.05).

**Table 4:** Qualitatively determined antibacterial activity of AS extracts obtained by ultrasonic extraction (UE), Soxhlet extraction (SE), and supercritical fluid extraction (SFE) on selected bacteria and fungi.

Microorganism		Inhibition Zone (mm ± SD)		
		UE (H_2_O)	SE (EtOH)	SFE (scCO_2_ + EtOH)
Gram-negative bacteria	*E. coli*	14 ± 2	17 ± 2	11 ± 1
	*P. aeruginosa*	11 ± 1	11 ± 1	11 ± 1
	*P. fluorescens*	15 ± 1	18 ± 2	15 ± 1
Gram-positive bacteria	*B. cereus*	-	15 ± 1	17 ± 1
	*S. aureus*	11 ± 1	13 ± 1	12 ± 1
	*S. platensis*	12 ± 1	11 ± 1	-
	*S. pyogenes*	-	11 ± 1	-
Fungi	*A. brasiliensis*	-	-	18 ± 3
	*A. flavus*	-	-	14 ± 1
	*A. fumigatus*	12 ± 1	-	22 ± 2
	*A. niger*	-	-	13 ± 2
	*C. albicans*	-	-	17 ± 2
	*P. cyclopium*	10 ± 1	13 ± 1	11 ± 1
	*S. cerevisiae*	-	-	-
	*T. viride*	-	-	-

**Table 5:** Minimum inhibitory concentrations (MIC_90_) for AS extracts obtained by ultrasonic extraction (UE; solvent H_2_O), Soxhlet extraction (SE; solvent EtOH), and supercritical fluid extraction (SFE; solvent scCO_2_, co-solvent EtOH) on various microorganisms.

Microorganisms		Incubation Period	MIC_90_ (μg/mL)		
			UE (H_2_O)	SE (EtOH)	SFE (scCO_2_ + EtOH)
Gram-negative bacteria	*E. coli*	8 h	-	280	280
		24 h	210	280	210
	*P. aeruginosa*	8 h	2780	280	140
		24 h	2780	280	280
	*P. fluorescens*	8 h	140	280	140
		24 h	280	2780	140
Gram-positive bacteria	*B. cereus*	8 h	70	2780	70
		24 h	280	2780	210
	*S. aureus*	8 h	280	140	140
		24 h	2780	210	280
	*S. pyogenes*	8 h	-	140	-
		24 h	2780	280	-
Fungi	*C. albicans*	8 h	-	-	2780
		24 h	-	-	2780

**Table 6:** Qualitative methods for phytochemical screening [94,95,96].

Phytochemicals	(Test) Procedure	Observation
Alkaloids	(Wagner’s reagent test) 1 mL of extract + 3–4 drops of Wagner’s reagent along the sides of test tube	A reddish-brown coloration
Anthocyanins	(NaOH test) 2 mL of extract + 1 mL of 2 N NaOH, heat for 5 min at 100 °C	A bluish-green color
Anthraquinones	(Borntrager’s test) 1 mL of extract + few drops of 10% (*w*/*v*) ammonia solution	Pink-colored solution
Carbohydrates	(Molish’s test) 2 mL of extracts + few drops of alcoholic α–naphthol + 1 mL conc. H_2_SO_4_ along the sides of test tube	Violet ring at the interface of the two liquids
Cardiac glycosides	(Keller–Killiani test) 1 mL of extract + 1.5 mL glacial acetic acid + 1 drop of 5% (*w*/*v*) FeCl_3_ solution + few drops of conc. H_2_SO_4_ along the side of test tube	A greenish-blue-colored solution
Coumarins	(NaOH test) 1 mL of 10% (*w*/*v*) NaOH + 1 mL of extract	A yellow color
Emodins	1 mL of extract + 2 mL NH_4_OH + 3 mL benzene	A red color
Flavonoids	(Alkaline reagent test) 1 mL of extract + 2 mL of 2% (*w*/*v*) NaOH + few drops of diluted HCl	A yellow color; solution then becomes colorless on addition of diluted acid
Phenolic compounds	(Ferric chloride test) 1 mL of extract + 2 mL of distilled H_2_O + few drops of 5% (*w*/*v*) FeCl_3_ solution	A blue/green/black color
Phlobatannins	(HCl test) 1 mL of extract + 2 mL of 1% (*v*/*v*) HCl	A red precipitate
Saponins	(Foam test) 2 mL of extract + 5 mL distilled H_2_O; vigorously shake for 15 min	Persistent foam after 10 min
Steroids	(Salkowski test) 2 mL of extract + 2 mL of CHCl_3_ + 2 mL of conc. H_2_SO_4_	A reddish-brown ring at the junction
Tannins	(Braymer’s test) 1 mL of extract + few drops of 10% (*w*/*v*) FeCl_3_ solution	A dark blue or greenish-black color
Terpenoids	1 mL of extract + 2 mL of CHCl_3_ + few drops (max. of 0.5 mL) of conc. H_2_SO_4_ along the side of test tube	A reddish-brown color at the interface
Quinones	(Sulfuric acid test) 1 mL of H_2_SO_4_ + 1 mL of extract; shake well for 5 min	A red color

## Data Availability

The data presented in this study are available in article.
